# Rosaceae fruit transcriptome database (ROFT)—a useful genomic resource for comparing fruits of apple, peach, strawberry, and raspberry

**DOI:** 10.1093/hr/uhad240

**Published:** 2023-11-14

**Authors:** Muzi Li, Stephen M Mount, Zhongchi Liu

**Affiliations:** Department of Cell Biology and Molecular Genetics, University of Maryland, College Park, MD 20742, USA; Department of Cell Biology and Molecular Genetics, University of Maryland, College Park, MD 20742, USA; Department of Cell Biology and Molecular Genetics, University of Maryland, College Park, MD 20742, USA

## Abstract

Rosaceae is a large plant family consisting of many economically important fruit crops including peach, apple, pear, strawberry, raspberry, plum, and others. Investigations into their growth and development will promote both basic understanding and progress toward increasing fruit yield and quality. With the ever-increasing high-throughput sequencing data of Rosaceae, comparative studies are hindered by inconsistency of sample collection with regard to tissue, stage, growth conditions, and by vastly different handling of the data. Therefore, databases that enable easy access and effective utilization of directly comparable transcript data are highly desirable. Here, we describe a database for comparative analysis, ROsaceae Fruit Transcriptome database (ROFT), based on RNA-seq data generated from the same laboratory using similarly dissected and staged fruit tissues of four important Rosaceae fruit crops: apple, peach, strawberry, and red raspberry. Hence, the database is unique in allowing easy and robust comparisons among fruit gene expression across the four species. ROFT enables researchers to query orthologous genes and their expression patterns during different fruit developmental stages in the four species, identify tissue-specific and tissue-/stage-specific genes, visualize and compare ortholog expression in different fruit types, explore consensus co-expression networks, and download different data types. The database provides users access to vast amounts of RNA-seq data across the four economically important fruits, enables investigations of fruit type specification and evolution, and facilitates the selection of genes with critical roles in fruit development for further studies.

## Introduction

Rosaceae is a large plant family consisting of over 3000 species. Many economically important fruits belong to this family including peach, apple, pear, strawberry, raspberry, plum, and others, and they are widely cultivated throughout the world. While past research has been largely focused on fruit ripening and quality traits [1], the early-stage fruit development is less well studied despite its complexity and critical importance. Early stage fruit development can be roughly divided into three phases, fruit set, cell division, and cell expansion [2]. In the first phase, successful pollination and fertilization trigger a specific floral tissue to commit to the development of a fruit. In the second phase, cell division is activated and fruit starts to enlarge. In the third phase, fruit size increases rapidly as a result of cell expansion and cell wall loosening. Investigations into early fruit development will help inform key developmental events and molecular mechanisms that underlie ultimate production of tasty, nutritious, and beautiful fruits.

In addition to its significant economic value, Rosaceae is an ideal family for investigations into molecular mechanisms underlying fruit type diversity [3, 4], featuring a large number of diverse fruit types including drupe (peach), pome (apple), drupetum (raspberry), and achenetum (strawberry). In drupe fruit, such as peach, the middle layer of the ovary wall (mesocarp) grows into fruit flesh, and the innermost layer of the ovary wall (endocarp) forms a hard stone encasing a single seed. In pome fruit (for example, apple), the majority fruit flesh is derived from the hypanthium, a cup-like structure outside the ovary wall. In drupetum and achenetum fruits, there are numerous individual ovaries sitting on the dome-like receptacle. In drupetum fruit like raspberry, each ovary develops into a fleshy drupelet. However, in achenetum fruit (strawberry), the receptacle becomes fleshy while the ovaries dry up to become the achenes dotting the receptacle fruit surface. As fruit tissue identity is likely determined during early stages of fruit development, comparative transcriptomic studies among different fruit types may shed light on mechanisms that endow the specific fruit types and evolution of different fruit types.

With the development of advanced high-throughput sequencing technologies, there has been dramatic increases in the number of chromosome-scale genome assemblies and comprehensive genome annotations for many Rosaceae species [5–10]. High-quality RNA-sequencing data for different Rosaceae fruits provide rich resources for investigations of plant growth and development, in particular, fruit development [11, 12]. The genomic data explosion also contributes to a greater number of databases established for economically important Rosaceae species [6]. For instance, the Genome Database for Rosaceae (GDR) [13] contains a large collection of Rosaceae genomes and integrates various tools for genetic and genomic analyses. *Fragaria vesca* co-expression network explorer (http://159.203.72.198:3838/fvesca) [14] offers consensus co-expression networks constructed to predict gene–gene relationships in strawberry. Furthermore, TRANSNAP [15] and strawberry eFP browser [16] (http://bar.utoronto.ca/efp_strawberry/cgi-bin/efpWeb.cgi) were developed for exploring the pear and strawberry transcriptome data, respectively. Although there is a variety of databases presenting different types of data from various Rosaceae species, few databases have been built for comparative analyses of transcriptome data among different Rosaceae species.

Here, we describe a comparative transcriptome database, ROFT, based on RNA-Seq data from four Rosaceae fruits, peach (*Prunus persica*), apple (*Malus x domestica*), wild strawberry (*Fragaria vesca*), and raspberry (*Rubus idaeus*), at early fruit developmental stages [11, 12, 17]. These fruits are of significant economic value, and each represents a unique fruit type, drupe, pome, achenetum, and drupetum, respectively. A comparative eFP browser in the database allows the users to compare gene expression patterns between orthologs in these four species. In addition, co-expressed genes and tissue-specific genes are provided for each of the four Rosaceae species to allow investigations of individual species or comparisons among the four species to gain a broader understanding of each gene. As a result, the common and distinct molecular features among the four fruit types can be identified, which may lead to a better understanding of early-stage fruit development and potential regulatory mechanisms underlying diverse fruit types and their development. The knowledge could lead to the design and creation of new fruit types that will enrich human diet and increase the diversity of fruit products. ROFT can be accessed at http://www.rosaceaefruits.com/.

## Results and discussion

### Summary of the ROFT database

**Figure 1:**
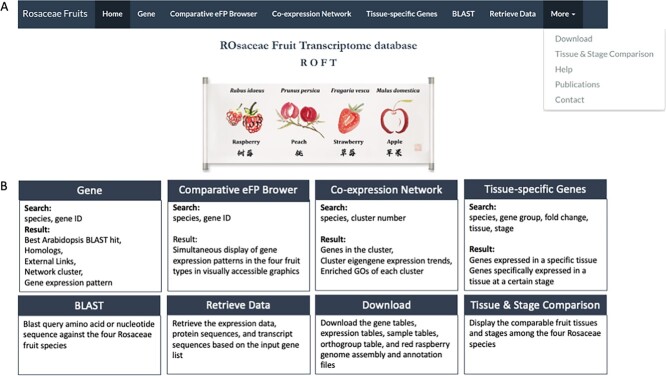
Summary of the ROFT Database (A) A screen shot of the ‘Home’ page tab. Note the tab bar on top showing different modules as tabs. (B) Summary of the key functions provided by each of the eight tabs.

**RO**saceae **F**ruit **T**ranscriptome database (ROFT), http://www.rosaceaefruits.com/, consists of seven main tabs, gene, comparative eFP browser, co-expression network, tissue-specific genes, BLAST, retrieve data, and more (Figure 1). Under the more tab, there are five subtabs, download, tissue & stage comparison, help, publications, and contact. Each tab and subtab are described in detail below.

### Gene

**Figure 2:**
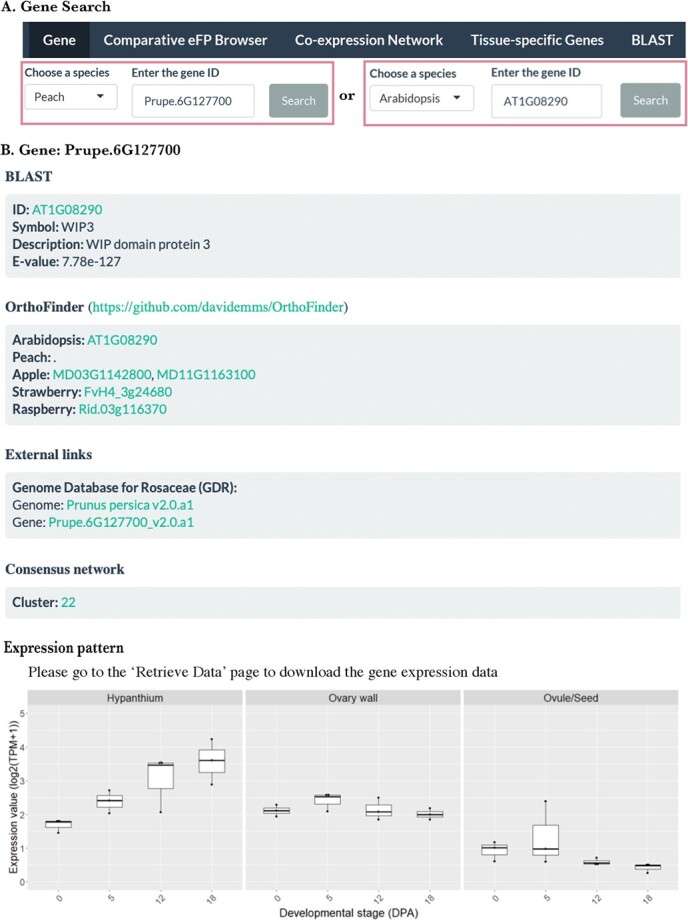
Illustration of the ‘Gene’ tab function. (A) Searching using the peach gene ID (left) or the Arabidopsis gene ID (right). *Prupe.6G127700* is the peach ortholog of the Arabidopsis gene *AT1G08290*. (B) The result from searching the peach gene shown in A. It provides information about the peach gene *Prupe.6G127700*, including its best BLAST hit in Arabidopsis, its Arabidopsis, apple, strawberry, and raspberry orthologs identified by OrthoFinder (https://github.com/davidemms/OrthoFinder), and external links to GDR regarding the specific genome assembly and gene of interest. Also shown are the consensus network cluster that this gene belongs to and the expression pattern for the gene of interest across the three fruit-related tissues at four early fruit developmental stages (Days Post Anthesis, DPA). Hyperlinks lead to additional information on the gene.

In the ‘gene’ tab, users can search for information on a gene from any of the four Rosaceae species, peach (*P. persica*), apple (*M. domestica*), wild strawberry (*F. vesca*), and red raspberry (*Rubus idaeus*) by entering in the search box a specific gene ID in the format of, for example, Prupe.2G047900 (peach), MD05G1107900 (apple), FvH4_2g03030 (strawberry), or Rid.02g042020 (raspberry) (Figure 2A). If such a gene ID is not known, one can use nucleotide or protein sequence of the gene to perform a blast using the BLAST function of ROFT, NCBI (https://blast.ncbi.nlm.nih.gov/Blast.cgi), or GDR (https://www.rosaceae.org/blast) to obtain the gene ID of the species of interest. The search result from ROFT-Gene tab includes the best BLAST hit in Arabidopsis (Arabidopsis gene ID, gene symbol, and gene description) as well as the query's orthologs (with specific gene IDs) in the four Rosaceae species (Figure 2B). The ‘orthologs’ refer to genes belonging to the same orthogroup identified by OrthoFinder [18].

Alternatively, one can use an Arabidopsis gene as a query (Figure 2A) to search for orthologs in the four Rosaceae species. The Arabidopsis locus ID can be obtained from TAIR (https://www.arabidopsis.org/). The search from ROFT using the Arabidopsis gene ID as a query similarly returns a description of the gene and a list of Rosaceae orthologs in the four species (Figure 2B). Hyperlinks are provided for each ortholog ID; clicking on one of the ortholog IDs will lead to the corresponding ‘gene’ page within the database showing its expression in fruit tissues and the co-expression cluster it belongs to. To learn more about the orthologous gene, one can click on the orthologous gene ID under external links, which will lead to GDR's gene page with a wealth of information about the gene including its coding sequence. For the Arabidopsis gene, one can click on the gene ID to access corresponding gene page in TAIR.

In addition, the ‘gene’ page indicates the consensus co-expression cluster number for the gene of interest (Figure 2B); clicking on the cluster number will lead to a consensus co-expression network page detailed in a later section. Finally, the ‘gene’ page provides the expression pattern for the gene of interest across three fruit-related tissues at four early fruit developmental stages (days post anthesis [DPA]) (Figure 2B).

### Comparative eFP (electronic fluorescent pictograph) browser

**Figure 3:**
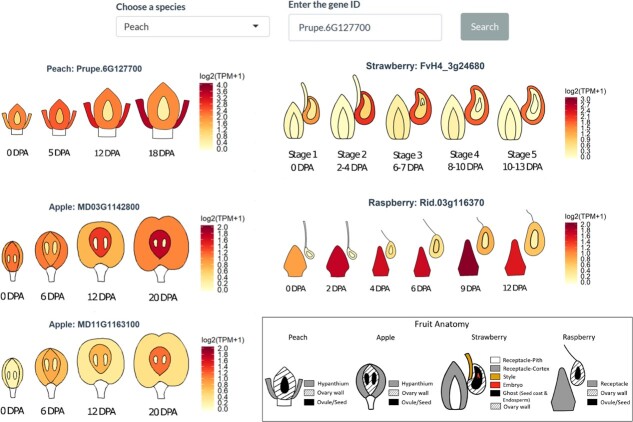
Comparative eFP browser showing the expression patterns of *WIP3* in fruit tissues of all four Rosaceae species. The peach *WIP3* gene (gene ID *Prupe.6G127700*) is entered here as a search example. There are two apple orthologs, and their expressions are both displayed. The scale bars are RNA-seq read levels expressed as log_2_(TPM + 1). The keys to the fruit tissue types are shown in the box, and specific fruit developmental stages as Days Post Anthesis (DPA) are shown beneath each stage. Sample description can be found in Table S1 and Figure S1.

In the ‘comparative eFP browser’ tab, one enters a specific gene ID of one of the Rosaceae species and hits ‘search’. The return is displayed as electronic fluorescent pictographs (eFPs) showing gene expression levels of all orthologs in apple, peach, strawberry, and raspberry. Hence, the user can simultaneously visualize and compare the gene expression patterns in botanically similar or distinct fruit tissues of the four Rosaceae species (Figure 3). If there are more than one ortholog, as is often the case for apple, the expression of all orthologs in a species will be shown. This comparative expression visualization makes it easier to compare and analyze gene expression differences between species and enables users to better illustrate the expression patterns and formulate novel hypothesis. A graphic key about different fruit tissues is provided (Figure 3). Table S1 provides details about the tissue samples from which RNAs were extracted.

### Co-expression network

Consensus co-expression network analyses were conducted for all four Rosaceae species [11, 12], providing rich information that can be mined from ROFT. For each of the four species, 1000 iterations of the WGCNA clustering [19] with each run resampling eighty percent of the genes led to a consensus correlation (co-efficient) matrix indicating the number of times a pair of genes were clustered together divided by the number of times they were subsampled together [14].

**Figure 4:**
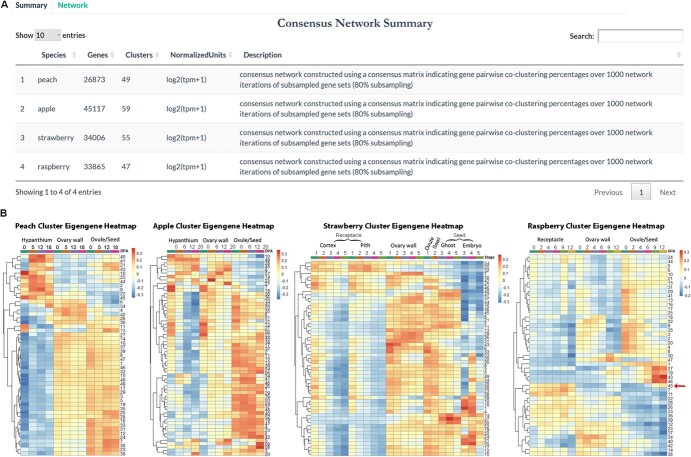
Illustration of the ‘summary’ subtab of the consensus ‘co-expression network’ tab. (A) Summary of consensus co-expression network analysis results showing the number of clusters in each species. (B) Heatmaps of cluster eigengene values, which provide the general expression trend of each cluster. An arrow points to cluster 45 of raspberry.

The ‘co-expression network’ tab includes two subtabs, ‘summary’ and ‘network’. The ‘summary’ subtab (Figure 4) provides basic statistics of the consensus co-expression networks including the total number of consensus co-expression clusters in each species, and a brief description of how the network was constructed (Figure 4A). Additionally, a heatmap illustrates the general expression trend of each cluster based on the eigengene value (Figure 4B). This provides users an overview of all the clusters in a species, based on which the users could choose a cluster of a specific expression trend for further exploration. For example, peach clusters 40, 41, 21, 43 all show preferential expression in the hypanthium and their expression appears post-fertilization (i.e. not expressed at 0 DPA). These four clusters might be of interest to users looking for fertilization induced gene expression changes specifically in the hypanthium.

**Figure 5:**
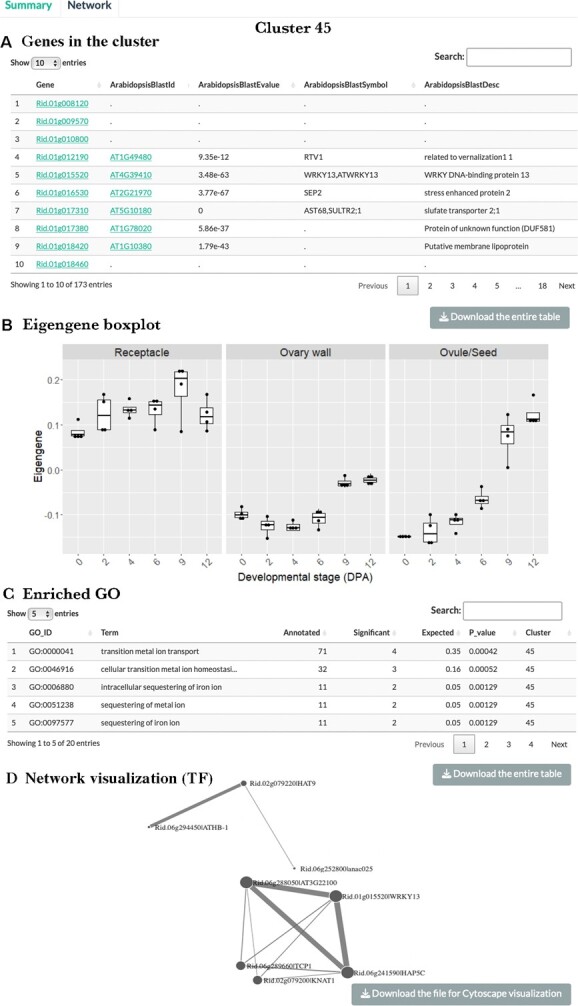
Illustration of the ‘network’ subtab of the consensus ‘co-expression network’ tab. By searching for raspberry cluster 45 in the search box, one can obtain a list of 173 raspberry genes belonging to cluster 45. Gene IDs are embedded with hyperlinks leading to information of respective genes in GDR or TAIR. The gene list can be downloaded by clicking the bottom right grey box. (B) Boxplots of cluster 45 eigengene values across raspberry fruit-related tissues and stages. Y-axis indicates the eigengene value, and X-axis indicates the developmental stages (Days Post Anthesis; DPA). (C) Top 20 enriched GO terms for cluster 45 downloadable by clicking the bottom right grey box. (D) Network visualization based on transcription factors (TFs) in the largest subnetwork (component) of cluster 45 with a correlation co-efficient >0.7. The visualization is interactive, where users can drag and rotate nodes (TF genes) and zoom in or out of the network. Edge thickness positively correlates with the correlation co-efficient between nodes; the node size positively correlates with the degree of connectivity (number of connections). For each cluster, a file containing correlations of all genes (TFs and non-TFs) with correlation co-efficient >0.7 in the largest subnetwork (component) of the cluster can be downloaded by clicking the bottom right grey box and explored further with Cytoscape.

In the ‘network’ subtab (Figure 5), one can select the species and then enter the cluster number in the search box. The search returns with a list of genes (gene ID, its best BLAST hit in Arabidopsis, and the description of the Arabidopsis gene) in the cluster of interest (see Figure 5A). The gene ID and its best Arabidopsis BLAST hit are clickable and linked to the external databases (GDR and TAIR). In addition, the subtab provides the cluster eigengene values across different fruit-related tissues and stages in a boxplot (Figure 5B), as well as the top 20 enriched GO terms for the cluster of interest (Figure 5C). The information will be helpful in identifying co-expressed genes in similar regulatory pathways and conducting comparative analyses between similar or contrasting clusters in different species.

To simplify the network for visualization, correlations among genes in a cluster with a correlation co-efficient equal or below 0.7 were removed. Consequently, not all genes are connected into a single network, leading to multiple subnetworks (components) each with subsets of the genes in a cluster. The subnetwork with the highest number of genes (nodes) in a cluster was saved and its corresponding correlation co-efficient file (containing both TFs and non-TFs) is downloadable by clicking the grey box ‘download the file for cytoscape visualization’. Users can open the file in cytoscape [21] for visualization and exploration of the network. In addition, we further filtered the file to save only transcription factors (TFs) with a correlation co-efficient >0.7, and the resulting file was used to construct an interactive network with the R package networkD3 [20] and displayed visually in the ‘network’ subtab (Figure 5D). Here, users not only can see connections among the nodes (TF genes) in the largest subnetwork (component) of a cluster but also can drag and rotate the nodes as well as zoom in or out of the network to gain understanding of these connections.

### Tissue-specific genes

The ‘Tissue-specific Genes’ tab, available for all four species, allows one to search for genes that are specifically expressed in a tissue at certain stage (select Tissue&Stage-Enriched in the pull-down menu) or expressed in a specific tissue (select the Tissue-Enriched in the pull-down menu) at multiple stages. In this second option, the output table lists the tissue-specific expression in various combinations of stages. Further, one can select 2-fold or 5-fold enrichment under ‘Minimum Fold Change’, which identifies genes with at least 2-fold or 5-fold higher expression in the selected tissue-stage than all other tissues and stages. Hence, 2-fold is less stringent than the 5-fold criterium and yields more genes. Finally, users can hit the ‘Download the entire table’ beneath the table to obtain an excel file which provides expression value of tissue-specific genes in all samples.

**Figure 6:**
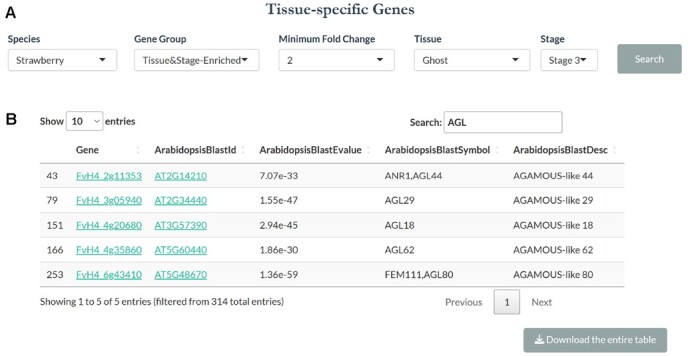
Demonstration of the ‘Tissue-Specific Genes’ tab. (A) The example search is in strawberry for genes specifically expressed in the ‘Ghost’ at stage 3 with a minimum of 2-fold enrichment. (B) The resulting gene list from search shown in A, showing a list of strawberry genes enriched specifically in the ghost tissue at stage 3 that includes several MADS-box genes. Note the clickable gene IDs and the ‘Download the entire table’ button beneath the table.

As illustrated in Figure 6, users first select strawberry under ‘species’ and then tissue&stage-enriched under ‘gene group’ from respective pull-down menus. Then, users select 2-fold enrichment under ‘minimum fold change’. Finally, users select Ghost under ‘tissue’ and Stage 3 under ‘stage’. Upon hitting ‘search’, it returns a table containing 314 genes that are at least 2-fold higher in expression in stage 3 ghost than all other tissues and stages. In the ghost tissue (seedcoat and endosperm) at stage 3 (6–7 DPA), which is a stage soon after fertilization, the identified genes are likely induced by fertilization specifically in the endosperm/seedcoat. Among these genes are several MADS-box genes including *AGL62* and *AGL80*. By clicking ‘Download the entire table’ grey box beneath the table, users will obtain the entire list of 314 genes and their expression levels, providing candidate genes for further functional studies.

### Blast

A BLAST function is included in the ROFT database. The users should select a particular Rosaceae species, and the blast will return the top blast hits of the specified species. Four BLAST programs, BLASTP, BLASTN, BLASTX, and TBLASTN, are available in the ‘BLAST’ section of ROFT.

### Retrieve data

This is another useful feature of ROFT. Under the data type's pull-down menu, one can select ‘expression data (TPM)’ to retrieve RNA-seq data (in transcripts per million, TPM) in different tissues and stages, or select ‘protein sequence data’ to obtain a gene’s protein sequence in FASTA format, or select ‘transcript sequence data’ to obtain a gene's full transcript sequence (5UTR + CDS + 3'UTR) of all isoforms. Users enter the gene ID in the ‘Retrieve Data’ section. Multiple gene IDs can be entered into the search box with one gene per line to retrieve sequence information of multiple genes.

### More: download, tissue & stage comparison, help, publications, and contact

In the ‘download’ tab accessible via ‘more’ tab, users can download the gene tables, expression tables, and sample tables for all genes in the genome of each of the four species. The gene table of each species includes the BLAST search results of all genes in the genome; for each gene, orthologs in Arabidopsis as well as the four Rosaceae species are provided as are gene description and membership in a specific consensus co-expression cluster. The expression table contains the gene expression level (TPM) across different floral/fruit tissues during fruit development. And the sample table (Table S1) describes in details the samples and stages from which the RNAs were isolated. The orthogroup table purely summarizes the orthologs among the Rosaceae species in comparison to Arabidopsis. In addition, users can download the red raspberry (*R. idaeus*) genome assembly and annotation files in the ‘download’ section.

In the ‘tissue & stage comparison’ tab accessible via ‘more’, diagrams are provided to illustrate the fruit tissues sampled in each species to aid in comparisons of equivalent tissues across the four species (Figure S1A). Further, a table (Figure S1B) is provided to illustrate comparable stages in the four species based on days post-anthesis (DPA) and embryo morphology during stages of fruit development. This information helps users to determine which tissue and which stage are comparable between species. As all samples of the four species were characterized, dissected, sequenced, and analyzed by the same laboratory, our data at ROFT facilitates robust comparative studies by minimizing inconsistencies in sample collection and data handling.

A ‘help’ page is also accessible through ‘more’ tab. This page aims to assist the users to take full advantage of the database. The ‘help’ section is composed of two components, ‘Q&A’ and ‘how-to videos’. The ‘Q&A’ mainly answers anticipated technical questions while the ‘how-to videos’ part is a screen recording tutorial video to demonstrate how one can explore various functions of the database.

Finally, a ‘publication’ page via ‘more’ tab lists all relevant publications based on which this database is built.

### Case study 1: *WIP3*, a likely conserved repressor of fleshy fruit development in Rosaceae species

*WIP* genes encode A1d subclass zinc-finger transcription factors known to inhibit plant organ development in different species including *Cucumis melo* [22], *Gerbera hybrida* [23], and Arabidopsis [24]. The protein is named due to the three conserved amino acids, tryptophan (W), isoleucine (I), and proline (P) at the C-terminus. The expression of *WIP* was examined in the four *Rosaceae* species by mining ROFT. First, the Rosaceae *WIP3* genes were identified by searching in the ‘gene’ section using the Arabidopsis *WIP3* gene (*AT1G08290*) as a query (Figure 2A). The search result lists *WIP3* orthologs in Arabidopsis, peach, apple, strawberry, and raspberry under OrthoFinder (Figure 2B). It shows two *WIP3* orthologs in apple, and one *WIP3* each in strawberry, raspberry, and peach.

Second, using the comparative eFP browser in ROFT, we discovered that *WIP3* showed extremely interesting expression patterns in the fruits of all four species. Specifically, in all cases, *WIP3* expression is low in tissues from which the fruit will develop, but higher in the non-fruit tissues (Figure 3), despite the fact that these are distinct tissues in the four species. For example, *WIP3* is relatively highly expressed in peach hypanthium, apple ovary wall, red raspberry receptacle, and strawberry ovary wall, tissues that are not forming fruit. Therefore, *WIP3* could potentially encode a conserved repressor of fruit flesh development.

### Case study 2: Metal ion transport appears active in raspberry receptacle and post-fertilization seeds

Previously, we showed that iron can travel from the receptacle to the ghost (seed coat and endosperm) after fertilization in strawberry [14]. The iron transported to the ghost may serve as the cofactor for GA biosynthetic enzymes, GA20ox and GA3ox, which lead to GA synthesis required for strawberry receptacle fruit enlargement [17]. Therefore, we explored the red raspberry consensus co-expression network in ROFT to determine if such iron transport activity may be conserved in the red raspberry. First, through the co-expression network's ‘summary’ page, we identified raspberry cluster 45 that exhibits receptacle-enriched expression as well as fertilization-induced expression in seeds (see red arrow in Figure 4B). Further exploration of cluster 45 in the ‘network’ page revealed that the top-ranking enriched GO terms of cluster 45 are associated with metal ion transport and homeostasis (Figure 5C). Hence, similar to strawberry, red raspberry receptacle also appears to experience active iron transport.

### Case study 3: Multiple MADS-box transcription factors encoded by *AGL*s (*AGAMOUS-LIKE*) may regulate strawberry seed development immediately post-fertilization

Each strawberry seed could be manually dissected and separated into embryo and ghost (seed coat and endosperm) starting from stage 3, which allows for the identification of the ghost-specific genes involved in seed development. To investigate what genes might be induced by fertilization in the endosperm-an important tissue for auxin and GA synthesis to stimulate post-fertilization programs, we mined the ROFT database in the ‘Tissue-specific Genes’ tab. 314 genes were identified that fulfill the search criteria: species- strawberry, gene group-tissue&stage-enriched, fold change-2, tissue-ghost, stage-stage 3 (Figure 6). Among the 314 genes expressed in the stage 3 ghost are five MADS-box genes including *AGL44* (*FvH4_2g11353*), *AGL29* (*FvH4_3g05940*), *AGL18* (*FvH4_4g20680*), *AGL62* (*FvH4_4g35860*), and *AGL80* (*FvH4_6g43410*) (Figure 6B). The functions of these genes in early seed development can be further tested. Interestingly, an interaction between AGL62 (FvH4_2g03030) and AGL80 (FvH4_6g08460) was shown in strawberry, and their function in promoting auxin biosynthesis in the strawberry seed was validated recently [25]. Therefore, the ROFT database provides an important bioinformatic resource for users to identify candidate genes and formulate hypotheses for future functional tests.

## Materials and methods

### Data source and processing

Table S1 provides details about all fruit-related tissue samples from which RNAs were extracted. The RNA-Seq data of peach (*P. persica*) and apple (*M. domestica*) ‘Honeycrisp’ were generated from three fruit tissues (hypanthium, ovary wall and ovule/seed) at four early stages of fruit development (0 DPA, 5/6 DPA, 12 DPA, and 18/20 DPA) in triplicates. Tissue morphology, staging, and the RNA-Seq data were described previously [11]. The data were deposited at SRA with the accession number PRJNA661345.

The wild strawberry (*F. vesca*) ‘yellow wonder’ early fruit development was divided into five stages, stage 1 (0 DPA), stage 2 (2–4 DPA), stage 3 (6–7 DPA), stage 4 (8–10 DPA), and stage 5 (10–13 DPA) [26]. The strawberry fruit tissues including style, pith, cortex, ovary wall, and ovule/seed (ghost and embryo) were dissected and harvested at their corresponding stages [17]. Two biological replicates were prepared for RNA-Seq, which was deposited at SRA with accession number PRJNA187983.

Three fruit tissues (receptacle, ovary wall, and ovule/seed) were dissected at six early stages of fruit development (0 DPA, 2 DPA, 4 DPA, 6 DPA, 9 DPA, and 12 DPA) of red raspberry (*R. idaeus*) ‘Joan J’. [12]. The RNA-Seq data were collected from four biological replicates and were deposited at SRA with accession number PRJNA869453. Cutadapt (v2.8) [27] was used to trim the low-qualify bases (cutoff: 25) from the 3′ end of the red raspberry reads. Only the reads with a minimum length of 36 bp were retained for the downstream analyses.

Salmon (v0.11.2) [28] was applied to quantify the transcript levels for the four Rosaceae species. The peach, apple, strawberry, and raspberry reference transcripts were retrieved from GDR (*P. persica* Genome v2.0.a1, *Malus x domestica* GDDH13 Whole Genome v1.1, *F. vesca* Genome v4.0.a2, and *R. idaeus* Joan J Genome v2.0) [12, 29–32]. For index construction, k-mer size was set to 31, and -keepDuplicates was specified to keep identical sequences in the reference transcripts. And -seqBias was passed to the quantifier to correct the sequence-specific bias. Tximport (v1.10.1) [33] was further utilized to summarize the transcript abundance at gene level.

### Ortholog detection

BLAST (v2.5.0) [34] was employed to search the longest protein isoforms of the four Rosaceae species against Arabidopsis protein database generated using the longest peptides in TAIR10_pep_20101214 (https://www.arabidopsis.org/download_files/Proteins/TAIR10_protein_lists/TAIR10_pep_20101214). The protein sequences of the four Rosaceae species were derived from the same genome annotations used for transcript quantification. Only the best Arabidopsis BLAST hits with *E*-value less than ${10}^{-5}$ were presented in the ROFT database.

The genes in peach, apple, strawberry, red raspberry, black raspberry (GDR: *Rubus occidentalis* whole genome assembly v3.0) [35], and Arabidopsis were assigned to different orthogroups by OrthoFinder (v2.3.8) [18]. The longest protein isoforms from each species were fed into OrthoFinder for ortholog identification.

### Development of comparative eFP browser

The R package ggefp (https://github.com/hredestig/ggefp) was used to visualize the gene expression data (log_2_(TPM + 1)) of the four Rosaceae species. The fruit diagrams shown in the comparative eFP browser were first created by Adobe Illustrator. The line drawings (PS format) were further transformed to ggproto objects by ggplot2 [36] and saved in RDA files that would be used by ggefp as exhibits for data visualization (https://github.com/hredestig/ggefp/blob/master/etc/trace.R).

### Consensus network construction

A robust signed co-expression network was constructed using the consensus clustering approach for each Rosaceae species [11, 12, 14]. WGCNA (peach and apple: v1.68, strawberry and red raspberry: v1.70.3) [19] was run 1000 times with subsampled genes and randomized parameters. The gene expression value was presented in log_2_(TPM + 1). Genes with little variance (≤ 0.05) and zero median absolute deviation were excluded. The biweight midcorrelation method was chosen to measure the similarity between the expression values of each pair of genes. A weighted adjacency matrix was produced by dividing the number of times genes were clustered together by the number of times genes were subsampled together. The ultimate consensus network was built based on the adjacency matrix using WGCNA with power 6 and minModuleSize 10.

The consensus correlation co-efficient matrix was filtered to remove gene pairs with correlation co-efficient = or < 0.7. Then, the R package igraph (v1.5.1) [37] was used to generate a file containing gene to gene correlations in the largest component (subnetwork) among the available components of a cluster. This file is downloadable from the ‘Download the file for Cytoscape visualization’ grey box in the ‘network’ subtab and can be explored with Cytoscape [21]. To visualize the TFs in the largest component of the cluster, the file above was filtered further by saving only TFs identified based on the PlantTFDB (v5.0) *Arabidopsis thaliana* TF list [38]. The resulting TF correlation file is visually displayed in the ‘network’ subtab using the R package networkD3 (v0.4) [20].

The GO terms associated with peach and apple genes were obtained from GDR (Prunus_persica_v2.0.a1_gene_functions.txt and Malus_x_domestica_GDDH13_v1.1_interpro.txt) while the GO annotations of strawberry and red raspberry were conducted by OmicsBox (v1.2.4) [39]. The R package topGO (v2.34.0) [40] was applied to perform the GO enrichment analysis for each co-expression cluster. The significance of the enriched GO categories was determined by Fisher’s exact test. The *p* value threshold was 0.05.

### Identification of tissue-specific genes

A combination of a tissue and a stage was regarded as a condition. Average TPM values of biological replicates were calculated to show the gene expression in each tissue at each stage (at each condition), which were later used by TissueEnrich (v1.2.1) [41] to identify the tissue- and stage-specific genes (condition-enriched genes), as well as the tissue-specific genes (multiple-condition-enriched, but within the same tissue). A gene is considered to be a tissue- and stage-enriched gene if its TPM is no less than 1, and its expression at a particular condition is at least two or five-fold higher than that at other conditions. Moreover, a gene is considered as a tissue-specific gene if its TPM is no less than 1, its expression at a group of conditions (within the same tissue) is at least 2-fold or 5-fold higher than that at other conditions, and the gene is not counted as a tissue- and stage-specific gene.

### Database implementation

The R package mongolite (v2.7.0) [42] was used to import the data into or fetch the data from MongoDB, a NoSQL database platform. And the user interface of the database was built by the R package Shiny (v1.7.4) [43]. The system hosting the database was Ubuntu (v22.04). The BLAST tool (v2.5.0) [34] embedded in the database was installed locally.

## Supplementary Material

Web_Material_uhad240

## Data Availability

The SRA accession number of peach and apple early fruit RNA-Seq data is PRJNA661345. The RNA-Seq data of strawberry and red raspberry early fruit development can be found in SRA using accession numbers PRJNA187983 and PRJNA869453, respectively.
