# Supplementary material for: Rosaceae fruit transcriptome database (ROFT)—a useful genomic resource for comparing fruits of apple, peach, strawberry, and raspberry
Source: Hortic Res. 2023 Nov 14;10(12):uhad240. doi: 10.1093/hr/uhad240 (PMC10756754; doi:10.1093/hr/uhad240)
Supplement: Web_Material_uhad240 [file web_material_uhad240.docx]

# Supplementary Figure 1 and Table 1

# Title: Rosaceae Fruit Transcriptome Database (ROFT) – a useful genomic resource for comparing fruits of apple, peach, strawberry, and raspberry

Muzi Li, Stephen M. Mount, and Zhongchi Liu*

Dept. of Cell Biology and Molecular Genetics, University of Maryland, College Park, MD 20742

**F
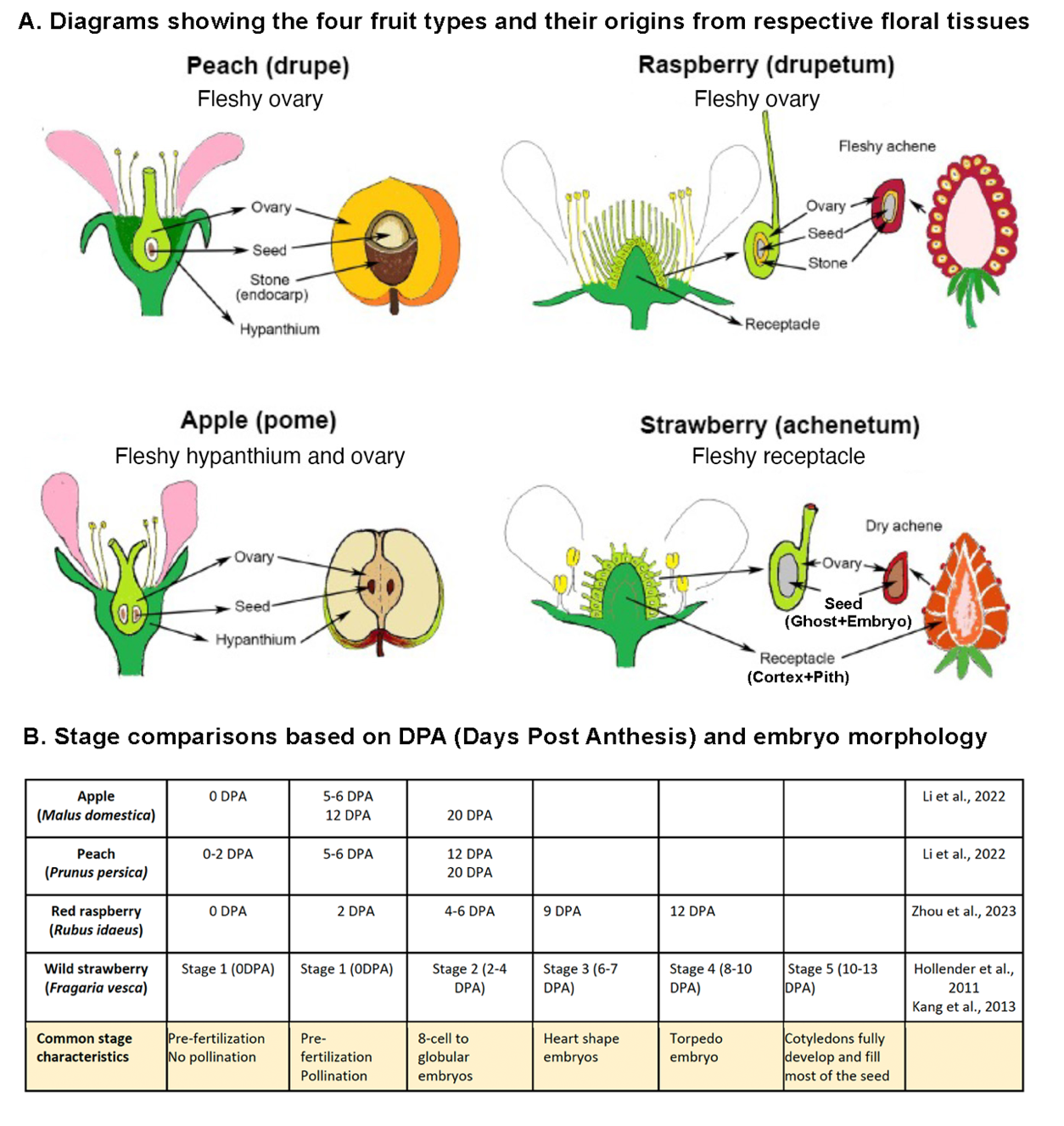
igure S1. Tissue and stage comparisons among apple, peach, strawberry and raspberry**

1. Diagrams showing the four fruit types and their origins from respective floral tissues. The fruit tissues used in this study include ovary wall, seed, hypanthium (peach and apple), and receptacle (strawberry and raspberry). Ovary illustrated here consists of ovary wall and the seed inside. The diagrams are based on Figure 1 of Liu et al., 2020.
2. Stage comparisons based on DPA (Days Post-Anthesis) and embryo morphology. The table is compiled based on prior studies ^1–4^.

Table S1 RNA-Seq samples generated from four Rosaceae species (strawberry, raspberry, peach and apple)

| **Strawberry (*F. vesca*)**  **hand dissected early-stage fruit tissues** ^2^ | |
| --- | --- |
| **TissueStage-Replicate No.** | **Sample description** |
| Cortex1-1 | Cortex of receptacle from just open flower, replicate 1 |
| Cortex1-2 | Cortex of receptacle from just open flower, replicate 2 |
| Cortex2-1 | Cortex of receptacle from the flowers which have been pollinated for about 3 days, replicate 1 |
| Cortex2-2 | Cortex of receptacle from the flowers which have been pollinated for about 3 days, replicate 2 |
| Cortex3-1 | Cortex of receptacle at about 6 DPA, same age as embryo-3, replicate 1 |
| Cortex3-2 | Cortex of receptacle at about 6 DPA, same age as embryo-3, replicate 2 |
| Cortex4-1 | Cortex of receptacle at about 9 DPA, same age as embryo-4, replicate 1 |
| Cortex4-2 | Cortex of receptacle at about 9 DPA, same age as embryo-4, replicate 2 |
| Cortex5-1 | Cortex of receptacle at about 12 DPA, same age as embryo-5, replicate 1 |
| Cortex5-2 | Cortex of receptacle at about 12 DPA, same age as embryo-5, replicate 2 |
| Pith1-1 | Pith of receptacle from just open flower, replicate 1 |
| Pith1-2 | Pith of receptacle from just open flower, replicate 2 |
| Pith2-1 | Pith of receptacle from the flowers which have been pollinated for about 3 days, replicate 1 |
| Pith2-2 | Pith of receptacle from the flowers which have been pollinated for about 3 days, replicate 2 |
| Pith3-1 | Pith of receptacle at about 6 DPA, same age as embryo-3, replicate 1 |
| Pith3-2 | Pith of receptacle at about 6 DPA, same age as embryo-3, replicate 2 |
| Pith4-1 | Pith of receptacle at about 9 DPA, same age as embryo-4, replicate 1 |
| Pith4-2 | Pith of receptacle at about 9 DPA, same age as embryo-4, replicate 2 |
| Pith5-1 | Pith of receptacle at about 12 DPA, same age as embryo-5, replicate 1 |
| Pith5-2 | Pith of receptacle at about 12 DPA, same age as embryo-5, replicate 2 |
| Ovary wall1-1 | Carpel walls (achene walls) from just open flower, replicate 1 |
| Ovary wall1-2 | Carpel walls (achene walls) from just open flower, replicate 2 |
| Ovary wall2-1 | Carpel walls (achene walls) from the flowers which have been pollinated for about 3 days, replicate 1 |
| Ovary wall2-2 | Carpel walls (achene walls) from the flowers which have been pollinated for about 3 days, replicate 2 |
| Ovary wall3-1 | Carpel walls (achene walls) at about 6 DPA, same age as embryo-3, replicate 1 |
| Ovary wall3-2 | Carpel walls (achene walls) at about 6 DPA, same age as embryo-3, replicate 2 |
| Ovary wall4-1 | Carpel walls (achene walls) at about 9 DPA, same age as embryo-4, replicate 1 |
| Ovary wall4-2 | Carpel walls (achene walls) at about 9 DPA, same age as embryo-4, replicate 2 |
| Ovary wall5-1 | Carpel walls (achene walls) at about 12 DPA, same age as embryo-5, replicate 1 |
| Ovary wall5-2 | Carpel walls (achene walls) at about 12 DPA, same age as embryo-5, replicate 2 |
| Ovule1-1 | Unfertilized Ovules from just open flower, replicate 1 |
| Ovule1-2 | Unfertilized Ovules from just open flower, replicate 2 |
| Seed2-1 | Seeds from the flowers which have been pollinated for about 3 days, replicate 1 |
| Seed2-2 | Seeds from the flowers which have been pollinated for about 3 days, replicate 2 |
| Ghost3-1 | Seeds without embryos inside (ie. endosperm + embryo) at about 6 DPA, same age as embryo-3, replicate 1 |
| Ghost3-2 | Seeds without embryos inside (ie. endosperm + embryo) at about 6 DPA, same age as embryo-3, replicate 2 |
| Ghost4-1 | Seeds without embryos inside (ie. endosperm + embryo) at about 9 DPA, same age as embryo-4, replicate 1 |
| Ghost4-2 | Seeds without embryos inside (ie. endosperm + embryo) at about 9 DPA, same age as embryo-4, replicate 2 |
| Ghost5-1 | Seeds without embryos inside (ie. endosperm + embryo) at about 12 DPA, same age as embryo-5, replicate 1 |
| Ghost5-2 | Seeds without embryos inside (ie. endosperm + embryo) at about 12 DPA, same age as embryo-5, replicate 2 |
| Embryo3-1 | Heart stage embryos, at about 6 DPA, replicate 1 |
| Embryo3-2 | Heart stage embryos, at about 6 DPA, replicate 2 |
| Embryo4-1 | Immature cotyledon stage embryos, at about 9 DPA, replicate 1 |
| Embryo4-2 | Immature cotyledon stage embryos, at about 9 DPA, replicate 2 |
| Embryo5-1 | Mature embryos which fill up entire seed, at about 12 DPA, replicate 1 |
| Embryo5-2 | Mature embryos which fill up entire seed, at about 12 DPA, replicate 2 |
| Style1-1 | Style from just open flower, replicate 1 |
| Style1-2 | Style from just open flower, replicate 2 |
| Style2-1 | Style from the flowers which have been pollinated for about 3 days, replicate 1 |
| Style2-2 | Style from the flowers which have been pollinated for about 3 days, replicate 2 |
|  |  |
| **Red Raspberry (*Rubus idaeus*)**  **Hand-dissected early-stage fruit tissues** ^4^ | |
| **Tissue (DPA)-sample No.** | **Sample description** |
| Receptacle (0DPA)-17 | Entire receptacle with achenes removed at the day when flower just opens, replicate 1 |
| Receptacle (0DPA)-27 | Entire receptacle with achenes removed at the day when flower just opens, replicate 2 |
| Receptacle (0DPA)-41 | Entire receptacle with achenes removed at the day when flower just opens, replicate 3 |
| Receptacle (0DPA)-S1 | Entire receptacle with achenes removed at the day when flower just opens, replicate 4 |
| Receptacle (2DPA)-1 | Entire receptacle with achenes removed at the day 2 after pollination, replicate 1 |
| Receptacle (2DPA)-17 | Entire receptacle with achenes removed at the day 2 after pollination, replicate 2 |
| Receptacle (2DPA)-4 | Entire receptacle with achenes removed at the day 2 after pollination, replicate 3 |
| Receptacle (2DPA)-S20 | Entire receptacle with achenes removed at the day 2 after pollination, replicate 4 |
| Receptacle (4DPA)-1 | Entire receptacle with achenes removed at the day 4 after pollination, replicate 1 |
| Receptacle (4DPA)-11 | Entire receptacle with achenes removed at the day 4 after pollination, replicate 2 |
| Receptacle (4DPA)-7 | Entire receptacle with achenes removed at the day 4 after pollination, replicate 3 |
| Receptacle (4DPA)-S25 | Entire receptacle with achenes removed at the day 4 after pollination, replicate 4 |
| Receptacle (6DPA)-1 | Entire receptacle with achenes removed at the day 6 after pollination, replicate 1 |
| Receptacle (6DPA)-10 | Entire receptacle with achenes removed at the day 6 after pollination, replicate 2 |
| Receptacle (6DPA)-7 | Entire receptacle with achenes removed at the day 6 after pollination, replicate 3 |
| Receptacle (6DPA)-S23 | Entire receptacle with achenes removed at the day 6 after pollination, replicate 4 |
| Receptacle (9DPA)-1 | Entire receptacle with achenes removed at the day 9 after pollination, replicate 1 |
| Receptacle (9DPA)-11 | Entire receptacle with achenes removed at the day 9 after pollination, replicate 2 |
| Receptacle (9DPA)-7 | Entire receptacle with achenes removed at the day 9 after pollination, replicate 3 |
| Receptacle (9DPA)-S28 | Entire receptacle with achenes removed at the day 9 after pollination, replicate 4 |
| Receptacle (12DPA)-1 | Entire receptacle with achenes removed at the day 12 after pollination, replicate 1 |
| Receptacle (12DPA)-13 | Entire receptacle with achenes removed at the day 12 after pollination, replicate 2 |
| Receptacle (12DPA)-4 | Entire receptacle with achenes removed at the day 12 after pollination, replicate 3 |
| Receptacle (12DPA)-S4 | Entire receptacle with achenes removed at the day 12 after pollination, replicate 4 |
| Ovary wall (0DPA)-17 | Carpel walls (achene walls) from the flower which just opens, ovules have been removed, replicate 1 |
| Ovary wall (0DPA)-24 | Carpel walls (achene walls) from the flower which just opens, ovules have been removed, replicate 2 |
| Ovary wall (0DPA)-7 | Carpel walls (achene walls) from the flower which just opens, ovules have been removed, replicate 3 |
| Ovary wall (0DPA)-S2 | Carpel walls (achene walls) from the flower which just opens, ovules have been removed, replicate 4 |
| Ovary wall (2DPA)-1 | Carpel walls (achene walls) at about 2 DPA (days post anthesis), seeds have been removed, replicate 1 |
| Ovary wall (2DPA)-17 | Carpel walls (achene walls) at about 2 DPA (days post anthesis), seeds have been removed, replicate 2 |
| Ovary wall (2DPA)-4 | Carpel walls (achene walls) at about 2 DPA (days post anthesis), seeds have been removed, replicate 3 |
| Ovary wall (2DPA)-S7 | Carpel walls (achene walls) at about 2 DPA (days post anthesis), seeds have been removed, replicate 4 |
| Ovary wall (4DPA)-1 | Carpel walls (achene walls) at about 4 DPA (days post anthesis), seeds have been removed, replicate 1 |
| Ovary wall (4DPA)-11 | Carpel walls (achene walls) at about 4 DPA (days post anthesis), seeds have been removed, replicate 2 |
| Ovary wall (4DPA)-7 | Carpel walls (achene walls) at about 4 DPA (days post anthesis), seeds have been removed, replicate 3 |
| Ovary wall (4DPA)-S24 | Carpel walls (achene walls) at about 4 DPA (days post anthesis), seeds have been removed, replicate 4 |
| Ovary wall (6DPA)-1 | Carpel walls (achene walls) at about 6 DPA (days post anthesis), seeds have been removed, replicate 1 |
| Ovary wall (6DPA)-10 | Carpel walls (achene walls) at about 6 DPA (days post anthesis), seeds have been removed, replicate 2 |
| Ovary wall (6DPA)-7 | Carpel walls (achene walls) at about 6 DPA (days post anthesis), seeds have been removed, replicate 3 |
| Ovary wall (6DPA)-S21 | Carpel walls (achene walls) at about 6 DPA (days post anthesis), seeds have been removed, replicate 4 |
| Ovary wall (9DPA)-1 | Carpel walls (achene walls) at about 9 DPA (days post anthesis), seeds have been removed, replicate 1 |
| Ovary wall (9DPA)-11 | Carpel walls (achene walls) at about 9 DPA (days post anthesis), seeds have been removed, replicate 2 |
| Ovary wall (9DPA)-7 | Carpel walls (achene walls) at about 9 DPA (days post anthesis), seeds have been removed, replicate 3 |
| Ovary wall (9DPA)-S26 | Carpel walls (achene walls) at about 9 DPA (days post anthesis), seeds have been removed replicate 4 |
| Ovary wall (12DPA)-1 | Carpel walls (achene walls) at about 12 DPA (days post anthesis), seeds have been removed, replicate 1 |
| Ovary wall (12DPA)-13 | Carpel walls (achene walls) at about 12 DPA (days post anthesis), seeds have been removed, replicate 2 |
| Ovary wall (12DPA)-4 | Carpel walls (achene walls) at about 12 DPA (days post anthesis), seeds have been removed, replicate 3 |
| Ovary wall (12DPA)-S5 | Carpel walls (achene walls) at about 12 DPA (days post anthesis), seeds have been removed, replicate 4 |
| Ovule (0DPA)-26 | Unfertilized ovules dissected out of the achenes in flowers that just open, replicate 1 |
| Ovule (0DPA)-41 | Unfertilized ovules dissected out of the achenes in flowers that just open, replicate 2 |
| Ovule (0DPA)-7 | Unfertilized ovules dissected out of the achenes in flowers that just open, replicate 3 |
| Ovule (0DPA)-S3 | Unfertilized ovules dissected out of the achenes in flowers that just open, replicate 4 |
| Seed (2DPA) -1 | Seeds dissected out of the achenes at about 2 DPA (days post anthesis), replicate 1 |
| Seed (2DPA)-17 | Seeds dissected out of the achenes at about 2 DPA (days post anthesis), replicate 2 |
| Seed (2DPA)-4 | Seeds dissected out of the achenes at about 2 DPA (days post anthesis), replicate 3 |
| Seed (2DPA)-S19 | Seeds dissected out of the achenes at about 2 DPA (days post anthesis), replicate 4 |
| Seed (4DPA) -1 | Seeds dissected out of the achenes at about 4 DPA (days post anthesis), replicate 1 |
| Seed (4DPA)-11 | Seeds dissected out of the achenes at about 4 DPA (days post anthesis), replicate 2 |
| Seed (4DPA)-7 | Seeds dissected out of the achenes at about 4 DPA (days post anthesis), replicate 3 |
| Seed (4DPA)-S8 | Seeds dissected out of the achenes at about 4 DPA (days post anthesis), replicate 4 |
| Seed (6DPA) -1 | Seeds dissected out of the achenes at about 6 DPA (days post anthesis), replicate 1 |
| Seed (6DPA)-10 | Seeds dissected out of the achenes at about 6 DPA (days post anthesis), replicate 2 |
| Seed (6DPA)-7 | Seeds dissected out of the achenes at about 6 DPA (days post anthesis), replicate 3 |
| Seed (6DPA)-S22 | Seeds dissected out of the achenes at about 6 DPA (days post anthesis), replicate 4 |
| Seed (9DPA) -1 | Seeds dissected out of the achenes at about 9 DPA (days post anthesis), replicate 1 |
| Seed (9DPA)-11 | Seeds dissected out of the achenes at about 9 DPA (days post anthesis), replicate 2 |
| Seed (9DPA)-7 | Seeds dissected out of the achenes at about 9 DPA (days post anthesis), replicate 3 |
| Seed (9DPA)-S27 | Seeds dissected out of the achenes at about 9 DPA (days post anthesis), replicate 4 |
| Seed (12DPA) -1 | Seeds dissected out of the achenes at about 12 DPA (days post anthesis), replicate 1 |
| Seed (12DPA)-13 | Seeds dissected out of the achenes at about 12 DPA (days post anthesis), replicate 2 |
| Seed (12DPA)-7 | Seeds dissected out of the achenes at about 12 DPA (days post anthesis), replicate 3 |
| Seed (12DPA)-S6 | Seeds dissected out of the achenes at about 12 DPA (days post anthesis), replicate 4 |
|  |  |
| **Peach (*Prunus persica*)**  **Hand dissected early-stage fruit tissues** ^3^ | |
| **Tissue (DPA)-sample No.** | **Sample description** |
| Hypanthium (0DPA)-9 | Hypanthium dissected from flowers that just open, replicate 1 |
| Hypanthium (0DPA)-28 | Hypanthium dissected from flowers that just open, replicate 2 |
| Hypanthium (0DPA)-44 | Hypanthium dissected from flowers that just open, replicate 3 |
| Hypanthium (5DPA)-9 | Hypanthium dissected from pollinated flowers at 5 DPA (days post anthesis), replicate 1 |
| Hypanthium (5DPA)-28 | Hypanthium dissected from pollinated flowers at 5 DPA (days post anthesis), replicate 2 |
| Hypanthium (5DPA)-44 | Hypanthium dissected from pollinated flowers at 5 DPA (days post anthesis), replicate 3 |
| Hypanthium (12DPA)-9 | Hypanthium dissected from pollinated flowers at 12 DPA (days post anthesis), replicate 1 |
| Hypanthium (12DPA)-28 | Hypanthium dissected from pollinated flowers at 12 DPA (days post anthesis), replicate 2 |
| Hypanthium (12DPA)-44 | Hypanthium dissected from pollinated flowers at 12 DPA (days post anthesis), replicate 3 |
| Hypanthium (18DPA)-9 | Hypanthium dissected from pollinated flowers at 18 DPA (days post anthesis), replicate 1 |
| Hypanthium (18DPA)-28 | Hypanthium dissected from pollinated flowers at 18 DPA (days post anthesis), replicate 2 |
| Hypanthium (18DPA)-44 | Hypanthium dissected from pollinated flowers at 18 DPA (days post anthesis), replicate 3 |
| Ovary wall (0DPA)-9 | Ovary wall dissected from flowers that just open, ovule has been removed, replicate 1 |
| Ovary wall (0DPA)-28 | Ovary wall dissected from flowers that just open, ovule has been removed, replicate 2 |
| Ovary wall (0DPA)-44 | Ovary wall dissected from flowers that just open, ovule has been removed, replicate 3 |
| Ovary wall (5DPA)-9 | Ovary wall dissected from pollinated flowers at 5 DPA (days post anthesis), seeds have been removed, replicate 1 |
| Ovary wall (5DPA)-28 | Ovary wall dissected from pollinated flowers at 5 DPA (days post anthesis), seeds have been removed, replicate 2 |
| Ovary wall (5DPA)-44 | Ovary wall dissected from pollinated flowers at 5 DPA (days post anthesis), seeds have been removed, replicate 3 |
| Ovary wall (12DPA)-9 | Ovary wall dissected from pollinated flowers at 12 DPA (days post anthesis), seeds have been removed, replicate 1 |
| Ovary wall (12DPA)-28 | Ovary wall dissected from pollinated flowers at 12 DPA (days post anthesis), seeds have been removed, replicate 2 |
| Ovary wall (12DPA)-44 | Ovary wall dissected from pollinated flowers at 12 DPA (days post anthesis), seeds have been removed, replicate 3 |
| Ovary wall (18DPA)-9 | Ovary wall dissected from pollinated flowers at 18 DPA (days post anthesis), seeds have been removed, replicate 1 |
| Ovary wall (18DPA)-28 | Ovary wall dissected from pollinated flowers at 18 DPA (days post anthesis), seeds have been removed, replicate 2 |
| Ovary wall (18DPA)-44 | Ovary wall dissected from pollinated flowers at 18 DPA (days post anthesis), seeds have been removed, replicate 3 |
| Ovule (0DPA)-9 | Unfertilized ovules dissected out of the ovaries in flowers that just open, replicate 1 |
| Ovule (0DPA)-28 | Unfertilized ovules dissected out of the ovaries in flowers that just open, replicate 2 |
| Ovule (0DPA)-44 | Unfertilized ovules dissected out of the ovaries in flowers that just open, replicate 3 |
| Seed (5DPA)-9 | Seeds dissected out of the ovaries at about 5 DPA (days post anthesis), replicate 1 |
| Seed (5DPA)-28 | Seeds dissected out of the ovaries at about 5 DPA (days post anthesis), replicate 2 |
| Seed (5DPA)-44 | Seeds dissected out of the ovaries at about 5 DPA (days post anthesis), replicate 3 |
| Seed (12DPA)-9 | Seeds dissected out of the ovaries at about 12 DPA (days post anthesis), replicate 1 |
| Seed (12DPA)-28 | Seeds dissected out of the ovaries at about 12 DPA (days post anthesis), replicate 2 |
| Seed (12DPA)-44 | Seeds dissected out of the ovaries at about 12 DPA (days post anthesis), replicate 3 |
| Seed (18DPA)-9 | Seeds dissected out of the ovaries at about 18 DPA (days post anthesis), replicate 1 |
| Seed (18DPA)-28 | Seeds dissected out of the ovaries at about 18 DPA (days post anthesis), replicate 2 |
| Seed (18DPA)-44 | Seeds dissected out of the ovaries at about 18 DPA (days post anthesis), replicate 3 |
|  |  |
| **Apple (*Malus domestica*)**  **Hand dissected early-stage fruit tissues** ^3^ | |
| **Tissue (DPA)-replicate No.** | **Sample description** |
| Hypanthium (0DPA)-1 | Hypanthium dissected from flowers that just open, replicate 1 |
| Hypanthium (0DPA)-2 | Hypanthium dissected from flowers that just open, replicate 2 |
| Hypanthium (0DPA)-3 | Hypanthium dissected from flowers that just open, replicate 3 |
| Hypanthium (6DPA)-1 | Hypanthium dissected from pollinated flowers at 6 DPA (days post anthesis), replicate 1 |
| Hypanthium (6DPA)-2 | Hypanthium dissected from pollinated flowers at 6 DPA (days post anthesis), replicate 2 |
| Hypanthium (6DPA)-3 | Hypanthium dissected from pollinated flowers at 6 DPA (days post anthesis), replicate 3 |
| Hypanthium (12DPA)-1 | Hypanthium dissected from pollinated flowers at 12 DPA (days post anthesis), replicate 1 |
| Hypanthium (12DPA)-2 | Hypanthium dissected from pollinated flowers at 12 DPA (days post anthesis), replicate 2 |
| Hypanthium (12DPA)-3 | Hypanthium dissected from pollinated flowers at 12 DPA (days post anthesis), replicate 3 |
| Hypanthium (20DPA)-1 | Hypanthium dissected from pollinated flowers at 20 DPA (days post anthesis), replicate 1 |
| Hypanthium (20DPA)-2 | Hypanthium dissected from pollinated flowers at 20 DPA (days post anthesis), replicate 2 |
| Hypanthium (20DPA)-3 | Hypanthium dissected from pollinated flowers at 20 DPA (days post anthesis), replicate 3 |
| Ovary wall (0DPA)-1 | Ovary wall dissected from flowers that just open, ovule has been removed, replicate 1 |
| Ovary wall (0DPA)-2 | Ovary wall dissected from flowers that just open, ovule has been removed, replicate 2 |
| Ovary wall (0DPA)-3 | Ovary wall dissected from flowers that just open, ovule has been removed, replicate 3 |
| Ovary wall (6DPA)-1 | Ovary wall dissected from pollinated flowers at 6 DPA (days post anthesis), seeds have been removed, replicate 1 |
| Ovary wall (6DPA)-2 | Ovary wall dissected from pollinated flowers at 6 DPA (days post anthesis), seeds have been removed, replicate 2 |
| Ovary wall (6DPA)-3 | Ovary wall dissected from pollinated flowers at 6 DPA (days post anthesis), seeds have been removed, replicate 3 |
| Ovary wall (12DPA)-1 | Ovary wall dissected from pollinated flowers at 12 DPA (days post anthesis), seeds have been removed, replicate 1 |
| Ovary wall (12DPA)-2 | Ovary wall dissected from pollinated flowers at 12 DPA (days post anthesis), seeds have been removed, replicate 2 |
| Ovary wall (12DPA)-3 | Ovary wall dissected from pollinated flowers at 12 DPA (days post anthesis), seeds have been removed, replicate 3 |
| Ovary wall (20DPA)-1 | Ovary wall dissected from pollinated flowers at 20 DPA (days post anthesis), seeds have been removed, replicate 1 |
| Ovary wall (20DPA)-2 | Ovary wall dissected from pollinated flowers at 20 DPA (days post anthesis), seeds have been removed, replicate 2 |
| Ovary wall (20DPA)-3 | Ovary wall dissected from pollinated flowers at 20 DPA (days post anthesis), seeds have been removed, replicate 3 |
| Ovule (0DPA)-1 | Unfertilized ovules dissected out of the ovaries in flowers that just open, replicate 1 |
| Ovule (0DPA)-2 | Unfertilized ovules dissected out of the ovaries in flowers that just open, replicate 2 |
| Ovule (0DPA)-3 | Unfertilized ovules dissected out of the ovaries in flowers that just open, replicate 3 |
| Seed (6DPA)-1 | Seeds dissected out of the ovaries at about 6 DPA (days post anthesis), replicate 1 |
| Seed (6DPA)-2 | Seeds dissected out of the ovaries at about 6 DPA (days post anthesis), replicate 2 |
| Seed (6DPA)-3 | Seeds dissected out of the ovaries at about 6 DPA (days post anthesis), replicate 3 |
| Seed (12DPA)-1 | Seeds dissected out of the ovaries at about 12 DPA (days post anthesis), replicate 1 |
| Seed (12DPA)-2 | Seeds dissected out of the ovaries at about 12 DPA (days post anthesis), replicate 2 |
| Seed (12DPA)-3 | Seeds dissected out of the ovaries at about 12 DPA (days post anthesis), replicate 3 |
| Seed (20DPA)-1 | Seeds dissected out of the ovaries at about 20 DPA (days post anthesis), replicate 1 |
| Seed (20DPA)-2 | Seeds dissected out of the ovaries at about 20 DPA (days post anthesis), replicate 2 |
| Seed (20DPA)-3 | Seeds dissected out of the ovaries at about 20 DPA (days post anthesis), replicate 3 |

1 Hollender CA, Geretz AC, Slovin JP, Liu Z. Flower and early fruit development in a diploid strawberry, Fragaria vesca. *Planta* 2012; **235**: 1123–1139.

2 Kang C, Darwish O, Geretz A, Shahan R, Alkharouf N, Liu Z. Genome-Scale Transcriptomic Insights into Early-Stage Fruit Development in Woodland Strawberry Fragaria vesca. *Plant Cell* 2013; **25**: 1960–1978.

3 Li M, Galimba K, Xiao Y *et al.* Comparative transcriptomic analysis of apple and peach fruits: insights into fruit type specification. *Plant J* 2022; **109**: 1614–1629.

4 Zhou J, Li M, Li Y *et al.* Comparative analyses of red raspberry and strawberry fruit development reveal diverse mechanisms for different fruit types. *Plant Physiol* 2023.
